# Improvement of Endurance Based on Muscle Fiber-Type Composition by Treatment with Dietary Apple Polyphenols in Rats

**DOI:** 10.1371/journal.pone.0134303

**Published:** 2015-07-29

**Authors:** Wataru Mizunoya, Hideo Miyahara, Shinpei Okamoto, Mariko Akahoshi, Takahiro Suzuki, Mai-Khoi Q. Do, Hideaki Ohtsubo, Yusuke Komiya, Mu Lan, Toshiaki Waga, Akira Iwata, Koichi Nakazato, Yoshihide Ikeuchi, Judy E. Anderson, Ryuichi Tatsumi

**Affiliations:** 1 Department of Animal and Marine Bioresource Sciences, Graduate School of Agriculture, Kyushu University, Hakozaki, Fukuoka, Japan; 2 Fundamental Research Laboratory, Asahi Breweries, Ltd., Moriya, Ibaraki, Japan; 3 Department of Physical Therapy, Faculty of Comprehensive Rehabilitation, Osaka Prefecture University, Habikino, Osaka, Japan; 4 Department of Exercise Physiology, Graduate School of Health and Sport Sciences, Nippon Sport Science University, Fukasawa, Tokyo, Japan; 5 Department of Biological Sciences, Faculty of Science, University of Manitoba, Winnipeg, MB, Canada; University of Minnesota Medical School, UNITED STATES

## Abstract

A recent study demonstrated a positive effect of apple polyphenol (APP) intake on muscle endurance of young-adult animals. While an enhancement of lipid metabolism may be responsible, in part, for the improvement, the contributing mechanisms still need clarification. Here we show that an 8-week intake of 5% (w/w) APP in the diet, up-regulates two features related to fiber type: the ratio of myosin heavy chain (MyHC) type IIx/IIb and myoglobin protein expression in plantaris muscle of 9-week-old male Fischer F344 rats compared to pair-fed controls (*P* < 0.05). Results were demonstrated by our SDS-PAGE system specialized for MyHC isoform separation and western blotting of whole muscles. Animal-growth profiles (food intake, body-weight gain, and internal-organ weights) did not differ between the control and 5% APP-fed animals (*n* = 9/group). Findings may account for the increase in fatigue resistance of lower hind limb muscles, as evidenced by a slower decline in the maximum isometric planter-flexion torque generated by a 100-s train of electrical stimulation of the tibial nerve. Additionally, the fatigue resistance was lower after 8 weeks of a 0.5% APP diet than after 5% APP, supporting an APP-dose dependency of the shift in fiber-type composition. Therefore, the present study highlights a promising contribution of dietary APP intake to increasing endurance based on fiber-type composition in rat muscle. Results may help in developing a novel strategy for application in animal sciences, and human sports and age-related health sciences.

## Introduction

Muscle fiber type proportions are responsible for a variety of properties of skeletal muscle, including contractile (fast- and slow-twitch), metabolic (glycolytic and oxidative, low and high resistance to fatigue), and sensory (differential tasting-component contents and fat deposition; see Ref. [1]); therefore mechanisms that regulate these properties, and their manipulation are hot targets of research for human sports and health and meat-animal production. Despite knowledge of the impact of motor-nerve impulse frequency [2–6] and the complex transcriptional circuitry including peroxisome proliferator-activated receptor (PPAR) α, δ, PPARγ coactivator1 (PGC1), and miR208b/499 elements in muscle fibers [7–14], the molecular mechanisms modulating fiber-type composition are not clearly understood.

Recent studies identified a promising contribution by dietary intake of a food ingredient to a significant increase in muscle endurance of mice and rats. Murase et al. [15, 16] reported positive effects of a 10-wk intake of 0.2–0.5% (w/w, dry-weight) of a polyphenol-rich green tea extract (GTE) containing 81% catechin and 0.1% caffeine, on swimming/running times-to-exhaustion in 8-wk-old male BALB/c mice. The effects were dose dependent and accompanied by lower respiratory quotients, higher β-oxidation activity and lower malonyl-CoA content in the muscle. Together the changes indicated an increased use of lipid as an energy source in GTE-fed mice. Similar benefits were found for dietary apple polyphenol (APP), which was originally prepared from unripe apples (*Malus pumila cv*. *Fuji*) by Nikka Whisky Distilling (Chiba, Tokyo). APP is a mixture of polyphenols that consist of about 45% (w/w) of procyanidin dimers to pentadecamers, 25% phenolic acids (mainly chlorogenic acid), 10% phloretin glycosides (mainly phloridzin), 15% monomeric flavan-3-ols (mainly catechin monomer) and 5% other compounds (mainly, quercetin glycosides) [17]. Nakazato et al. [18] first showed that 3 weeks of APP feeding (5% w/w) significantly reduced the weight of retroperitoneal and epididymal adipose tissue and the proliferation of pre-adipocytes without changing plasma-lipid profiles of triglycerides, total cholesterols, and nonesterified fatty acids; that diet also did not change cumulative energy intake or body-weight gain in 11-wk-old male Wister rats compared to controls, suggesting an anti-adipogenic effect of dietary APP. Importantly, a subsequent study reported enhanced muscle contractility, in that the relative isometric twitch torque of the ankle-joint (normalized to initial torque) stayed higher in the 5% APP-fed group during a 2-min train of transdermal nerve stimulation (10 V, 1 Hz) with skin electrodes fixed on over the medial gastrocnemius (*P* < 0.05). Higher expression of PPARα and δ, positive regulators of lipid oxidation, after feeding with 5% APP suggested that enhanced lipid metabolism may contribute to increasing fatigue resistance (see Figs 4 and 5 in Ref. [19]).

In order to further evaluate the potential of dietary APP to improve muscle endurance, the present 8-wk pair-feeding experiment was designed precisely to examine changes in the levels of adult myosin heavy chain isoforms (MyHC types I, IIa, IIx, and IIb) and myoglobin (the primary oxygen-carrying pigment in vertebrate myofibers) in plantaris muscles of 9-wk-old male Fischer F344 rats. Respective measures by our modified gel electrophoresis (SDS-PAGE) system [20] and western blotting, were expected to provide direct indices of the essential biochemical and energetic properties responsible for changes in fiber-type composition and muscle endurance. Results clearly demonstrated that 8 wk of a 5%-APP diet significantly elevates the type-IIx/IIb ratio and the level of myoglobin protein (*P* < 0.05); the changes may explain the increased fatigue resistance that was revealed here by a slower decline in the maximum isometric planter-flexion torque generated by successive electrical-stimulation of tibial nerve.

## Materials and Methods

### Animals and pair-feeding

All animal experiments were conducted in strict accordance with the recommendations in the Guidelines for Proper Conduct of Animal Experiments published by the Science Council of Japan and ethics approval from the Kyushu University Institutional Review Board (approval no. A21-117, A22-148, and A24-138).

Male Fischer F344 rats (purchased from KBT Oriental, Tosu, Japan at 7-wks old) were housed at 22 ± 2°C and 55 ± 10% humidity on a 12:12 light/dark cycle and received water and AIN-93G diet *ad libitum* during a 2-wk equilibration period. Subsequently, rats were divided into two groups (n = 9/group) with no difference in body weight (169.6 ± 1.4 *vs*. 169.9 ± 1.8 g (mean ± SE)). The two groups were maintained for the next 8-wk period on an AIN-93G dry-powder diet, with or without 5% (w/w) APP by a pair-feeding method in which food intake (g) was adjusted to be equivalent between the control and 5% APP-fed groups. APP was supplied from Fundamental Research Laboratory, Asahi Breweries, Moriya, Ibaraki, Japan and the diet had the same dietary APP concentration (5%) and overall formulation (S1 Table) as that used in previous studies by Nakazato et al. [18, 19]. Note that APP was substituted with cornstarch in the control diet resulting in a 5% increase in carbohydrate content for the control group. Food intake and body weight were recorded daily for each animal. A separate, paired set of rats received either a 0.5% (w/w) APP or control diet, *ad libitum* for 8 wks starting at 9 wks-of-age to study effects of a low-dose-APP diet (*n* = 9 per group in control and 0.5%-APP groups, with initial body weight (mean ± SE) of 171.7 ± 1.1 *vs*. 171.6 ± 1.8 g, respectively).

Immediately after euthanasia by decapitation under intra-peritoneal (*ip*) sodium pentobarbital anesthesia (50 mg/kg of body weight), a variety of internal organs (liver, kidney, spleen, and heart) and lower hind limb muscles (soleus, plantaris, tibialis anterior (TA), gastrocnemius, and extensor digitorum longus (EDL)) were collected and weighed. Adipose tissues (epididymal, perinephric, mesenteric fats, and brown adipose) were also weighed for comparison of dietary APP effects on fat metabolism [18, 19]. Muscle samples were frozen in isopentane cooled with liquid nitrogen and stored (-80°C) until use for SDS-PAGE and western blotting. Plantaris muscles were additionally analyzed for total protein content (% dry weight) by a Dumatherm nitrogen-determination system run under the combustion method (Gerhardt Japan, Tokyo, Japan).

### Tetanic force measurements

**Fig 1:**
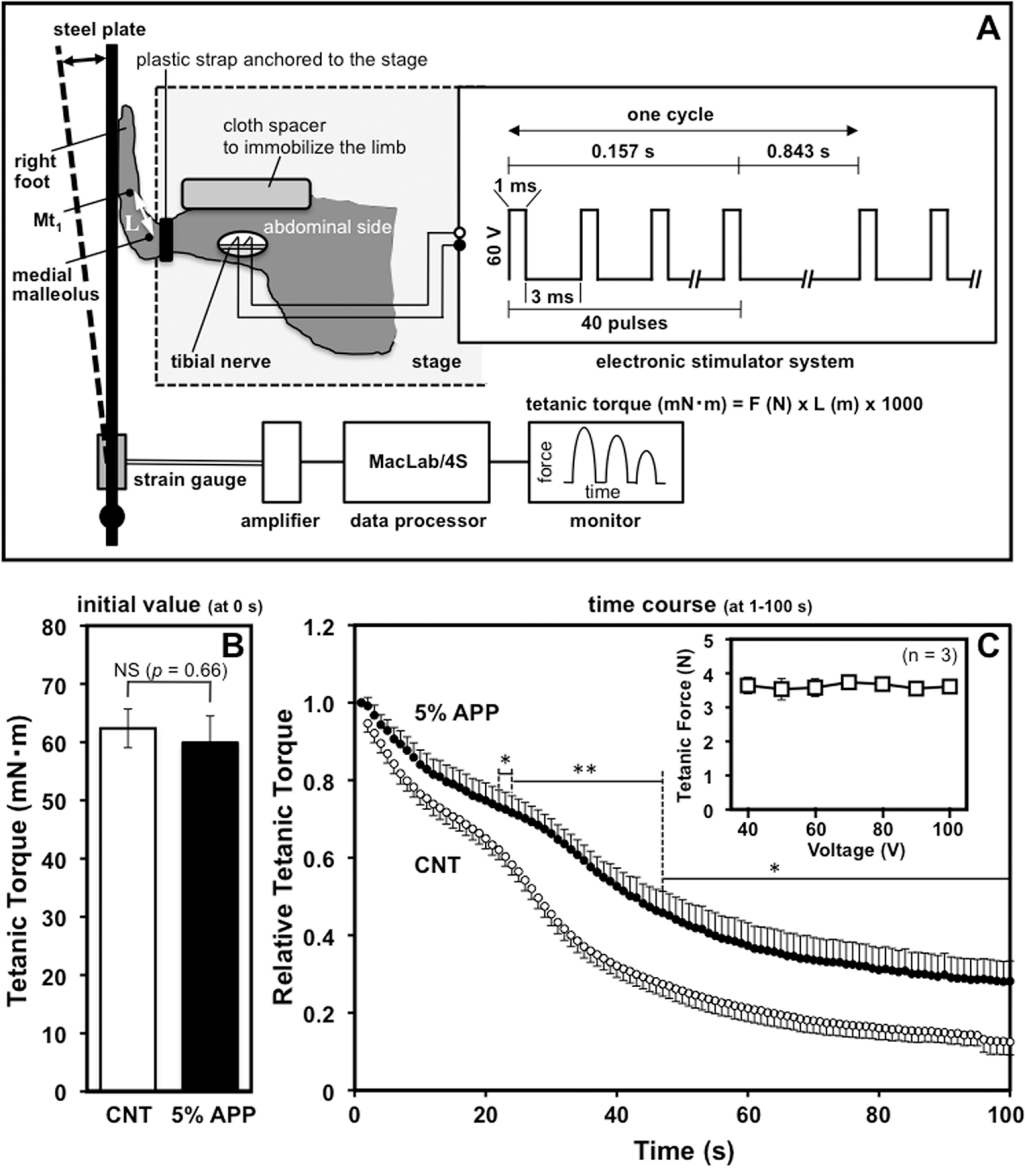
Improvement of muscle endurance by 5% APP feeding. Maximum isometric planter-flexion force torque was measured under anesthesia after 8-wk feeding of the control or 5%-APP diet, according to Iwata et al. [21] with some modifications. Schematic configuration of equipment is shown in panel A, in which strain gauges are attached to the footplate and connected to an instrumentation amplifier, a MacLab/4S digital converter, and a PowerMacintosh computer. A hook-shaped electrode connected to an electronic-stimulator module generated a stimulus train (250 Hz: pulse width 1 ms, duration 160 ms with intervals of 3 ms) delivered to the right tibial nerve branch that innervates calf muscles of the lower hind limb, including plantaris and gastrocnemius muscles. The amplitude (60V) was optimized as the supramaximal voltage by a separate voltage-dependency experiment using 3 control rats in which tetanic forces generated at the first electrical stimulus (at 0-s) were measured in a dose-range of 40–100 V (panel C inset). Tetanic-contraction forces measured were converted to torque (mN · m) by multiplying by the length (L) between the medial malleolus and the head of the first metatarsal bone (Mt_1_); the initial value at the first stimulation (at 0-s) and the time-course change in response to 100-s successive stimulation (expressed relative to the initial value) are shown in panels B and C, respectively. Data points and bars depict the mean ± SE for nine rats in each group fed with control (open circle and bar) or the 5%-APP diet (closed circle and bar) and significant differences from control at *P* < 0.05 and *P* < 0.01 are indicated by (*) and (**), respectively. NS, no significant difference.

After 8-wk feeding of the APP or control diet, maximum isometric planter-flexion force torque was measured under sodium pentobarbital anesthesia, according to Iwata et al. [21] with some modifications. Briefly, a set of four strain gauges (KFWS-2N-120-C1-11, Kyowa Electronic Instruments, Tokyo, Japan) attached to a steel footplate was connected to an instrumentation amplifier (WGA-101AS1; Kyowa Electronic Instruments) and a MacLab/4S digital converter (ADInstruments, Colorado Springs, CO, USA), as shown in Fig 1A. Tetanic contraction force of the posterior muscle in the lower hind limb (right side) was generated by successive electrical stimulations with an electronic stimulator module (SEN-5201 and SEN-3201; Nihon Kohden, Tokyo, Japan) via a bipolar hook-shaped electrode (KS207-024; Unique Medical, Tokyo, Japan) on tibial nerve. Force was recorded for 100 s using an Apple PowerMacintosh 7600/132 computer, and was converted to torque by multiplying the length between the medial malleolus and the head of the first metatarsal bone (Mt_1_) for that rat. The stimulation conditions (amplitude 60V, rectangular pulse width 1 ms, duration 160 ms with intervals of 3 ms after each pulse, frequency 250 Hz) were optimized to generate maximum isometric force at the initial electrical stimulation (see Fig 1C inset).

### SDS-PAGE of MyHC isoforms

Whole plantaris muscle samples (100 ng protein) were run on 8% polyacrylamide gels (99:1 of the acrylamide monomer/*N*,*N’*-methylenebisacrylamide ratio; 83 mm width x 73 mm length x 0.5 mm thickness of a Bio-Rad mini-gel dimension) under reducing conditions as described previously [20]. Briefly, frozen powdered samples were homogenized by a needle-type sonicator in a PRO-PREP protein extraction solution (17081, formulated with protease inhibitor cocktail containing PMSF, EDTA, pepstatin A, leupeptin, and aprotinin; iNtRON Biotechnology, Gyeonggi-do, Korea) followed by centrifugation at 13,000 rpm for 5 min; supernatants were diluted in 2x sample-buffer (4% (w/v) SDS, 100 mM dithiothreitol (DTT), 43% (v/v) glycerol, 0.16 M Tris-HCl buffer (pH 6.8), and 0.2% (w/v) bromophenol blue) to adjust to a final protein concentration of 20 μg/ml as assayed by the bicinchoninic acid (BCA) method standardized with bovine serum albumin (BSA). Samples were boiled for 3 min. The separating gel was composed of 8% polyacrylamide, 35% glycerol, 0.1 M glycine, 0.4% SDS, 0.2 M Tris-HCl (pH 8.8), 0.1% (w/v) ammonium persulfate (APS), and 0.05% (v/v) *N*,*N*,*N’*,*N’*-tetramethylethylenediamine (TEMED). The stacking gel consisted of 4% polyacrylamide (50:1), 30% glycerol, 4 mM EDTA, 0.4% SDS, 70 mM Tris-HCl (pH 6.7), 0.1% APS, and 0.05% TEMED. The lower running buffer (pre-cooled) consisted of 50 mM Tris (base), 75 mM glycine, and 0.05% SDS, while the upper buffer was at 6x the concentration of the lower buffer plus 0.12% (v/v) β-mercaptoethanol. Samples (5 μl) were run at a constant voltage of 140 V for about 23 h in an incubator set at 4°C with gentle stirring of the pre-cooled lower buffer; gels were silver stained (37937–96, Silver Stain KANTO III; Kanto Chemical, Tokyo Japan) followed by densitometry of MyHC isoforms using ImageJ 1.34 s software (originally developed by Dr. Wayne Rasband, National Institutes of Health, Bethesda, MD, USA). A typical separation pattern of MyHC isoforms provides high resolution of type IIa, IIx, IIb, and I isoforms (visualized in order from the cathode) in a mixed sample of soleus and EDL muscles, and thus ensures a qualitative advantage for densitometry of rodent MyHC isoforms.

### ECL-western blotting of myoglobin

Whole muscle samples (100 ng proteins) were subjected to 12.5% SDS-PAGE under reducing conditions and transferred onto Amersham Hybond ECL nitrocellulose membranes (RPN2020D; GE Healthcare, Little Chalfont, UK), as described previously [22]. The blots were blocked with 10% powdered milk in 0.1% (v/v) polyethylene sorbitan monolaurate (Tween 20)-Tris buffered saline (TTBS) before incubation with a mouse monoclonal primary antibody cocktail of anti-myoglobin (clone MG-1; M7773, Sigma, St. Louis, MO, USA; 1:1000 dilution) [23, 24] and anti-α-tubulin (clone DM1A; T9026, Sigma; 1:1000) [25, 26] in CanGetSignal solution 1 (NKB-101; Toyobo, Osaka, Japan) overnight at 4°C. Membranes were treated for 1 h with biotinylated anti-mouse IgG secondary antibody (BA-9200; Vector Labs., Burlingame, CA, USA) at 1:5000 dilution in CanGetSignal solution 2 (NKB-101; Toyobo) and with horseradish peroxidase (HRPO)-labeled avidin (PK-6100; Vector Labs.) at 1:500 dilution in TTBS for 30 min at room temperature, followed by enhanced chemiluminescence (ECL) detection (RPN2106; GE Healthcare) onto Amersham Hyperfilm ECL (28906837; GE Healthcare) according to the manufacturer’s recommendation. Myoglobin band intensity was quantified by densitometry normalized to α-tubulin as an internal standard.

### Real-time RT-qPCR and immuno-histochemistry

(for S6 and S7 Figs)

Total RNA was isolated from plantaris according to a regular Trizol-chloroform protocol. cDNA was synthesized from 2 μg of total RNA by a reverse-transcriptase SuperScript III (18080–044; Invitrogen, Grand Island, NY, USA) using oligo(dT) primer (H09876; Roche, Mannheim, Germany). The levels of mRNA expression of rat PGC1α (accession no. NM_031347) were monitored by real-time quantitative PCR (qPCR) using Roche LightCycler1.5 run under the TaqMan probe-detection format (Roche) standardized with the expression of hypoxanthine guanine phosphoribosyl transferase (HPRT; accession no. NM_012583.2). All primer sets were designed using a Roche Universal ProbeLibrary Assay Design Center (ProbeFinder version 2.35 for rat) with an intron-spanning assay: for PGC1α, forward 5’-gcagtcgcaacatgctca-3’, reverse 5’-gggtcatttggtgactctgg-3’ (amplicon 72 nt, Roche TaqMan Probe no. 6); for HPRT, forward 5’-gaccggttctgtcatgtcg-3’, reverse 5’-acctggttcatcatcactaatcac-3’ (amplicon 61 nt, Probe no. 95). Annealing temperature was set to 60°C in both cases.

In order to visualize slow fibers in plantaris muscle, cryosections (10 μm thick) were fixed with 3.7% paraformaldehyde in phosphate-buffered saline (PBS) and blocked with 3% BSA in TTBS followed by incubation in primary antibody to MyHC type-I (1:500 dilution; clone NOQ7.5.4D mouse monoclonal, M8421 from Sigma), HRPO-labeled secondary antibody (A4416; Sigma) and color development with 3,3’-diaminobenzidine (DAB) substrate. Sections were counter-stained with hematoxylin, mounted in Permount medium and observed using a Leica DMI6000B-AFC microscope equipped with a DFC295 digital color camera and LAS V4.5 controller.

### Statistical analysis

The Student’s *t-*test was employed for statistical analysis of experimental results using Excel X for Macintosh (Microsoft). Data are represented as mean ± SE for nine rats per treatment group. Statistically significant differences from the control group at *P* < 0.05 and *P* < 0.01 are indicated on graphs by (*) and (**), respectively. Each experiment was repeated independently, two or three times to verify the reproducibility of results.

## Results and Discussion

### Growth profiles of animals

The purpose of this study was to examine if fiber-type composition would be altered by feeding a 5% APP diet to 9-wk-old male Fischer F344 rats (*n* = 9 in each diet group). As shown in S1 Fig, under the 8-wk pair-feeding condition, the total dietary intake (panel A) and growth profiles for body weight (panel B) and internal organ weights (panel C; g/100 g body weight) were comparable for control and 5%-APP groups at the end of the experimental period. Additionally, APP significantly decreased the weight of adipose tissues including epididymal, perinephric, mesenteric fats, and brown adipose tissue (S2 Fig panel A; g/100 g body weight), consistent with a previous observation by Nakazato et al. [18]. Muscle weight was also slightly lower after 5%-APP feeding (*P* < 0.05 for gastrocnemius, EDL, and heart; see S1 Fig panel C and S2 Fig panel B), possibly due to higher intramuscular-fat metabolism rather than decreased protein intake as revealed by total nitrogen determination for plantaris muscle (% dry weight; see S2 Fig panel C). These measures provided the essential background assurance of reliable pair feeding.

### Increase in muscle endurance

The effects of a 5%-APP diet on muscle endurance were evaluated at the end of the 8-wk feeding period by comparing the time course of maximum isometric planter-flexion torque generated through 100-s of successive electrical stimulation (250 Hz: pulse width 1 ms, duration 160 ms with intervals of 3 ms) of tibial nerve bundles innervating the calf muscles of the lower hind limb (Fig 1). The nerve-stimulation protocol here is a refinement of the transdermal stimulation reported by Nakazato et al. [19] in which the net amplitude may vary depending on a variety of endogenous factors, including the skin thickness and subcutaneous fat amount that is presumably reduced in APP-fed animals; this approach contributes to a precise comparison of the maximum isometric planter-flexion torque between the control and APP-fed groups in the present study. The supramaximal amplitude (60 V) was also optimized here by a separate voltage-dependency experiment to show that tetanic forces generated at the first electrical stimulus (at 0-s) reached a plateau in a dose-range of 40–100 V (see panel C inset). As clearly demonstrated in panels B and C, while the initial tetanic torques (mN · m) at the first stimulation (at 0-s) were comparable between the control and 5% APP-diet groups (*P* = 0.66), the resistance to fatigue (torque expressed relative to the initial value) was significantly higher in the APP-fed group than the control which declined to less than 12% of the initial value at 100-s (IC_50% decrease_: about 43 *vs*. 28 s for 5% APP *vs*. control groups, respectively). This is direct evidence that 8 wks of 5%-APP feeding significantly improves muscle endurance of calf muscle of rats.

### Changes in fiber-type composition

**Fig 2:**
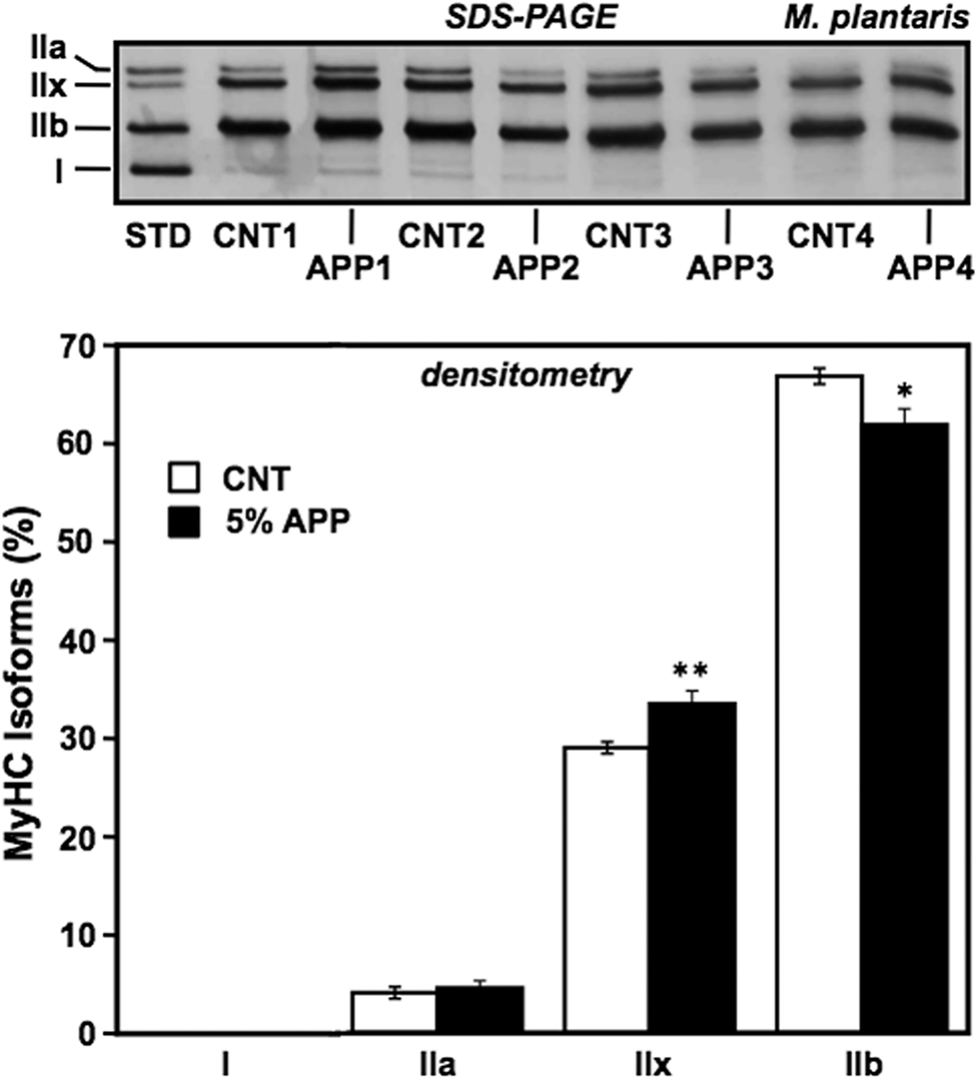
Increased MyHC IIx/IIb ratio by 5% APP feeding. Plantaris muscles from control and 5%-APP diet groups (after 8-wk feeding) were analyzed for each the ratio of each MyHC isoform (%) relative to total MyHC, by SDS-PAGE (upper panel; representative 4 samples from each group) and the densitometry (lower panel). STD, a mixed sample of soleus and EDL muscles from a control rat served as a standard that contains all adult-type MyHC isoforms, IIa, IIx, IIb, and I, in this order from the cathode. Data bars depict the mean ± SE for nine rats per group fed with control (open bar) or a 5%-APP diet (closed bar) and significant differences from control at *P* < 0.05 and *P* < 0.01 are indicated by (*) and (**), respectively.

**Fig 3:**
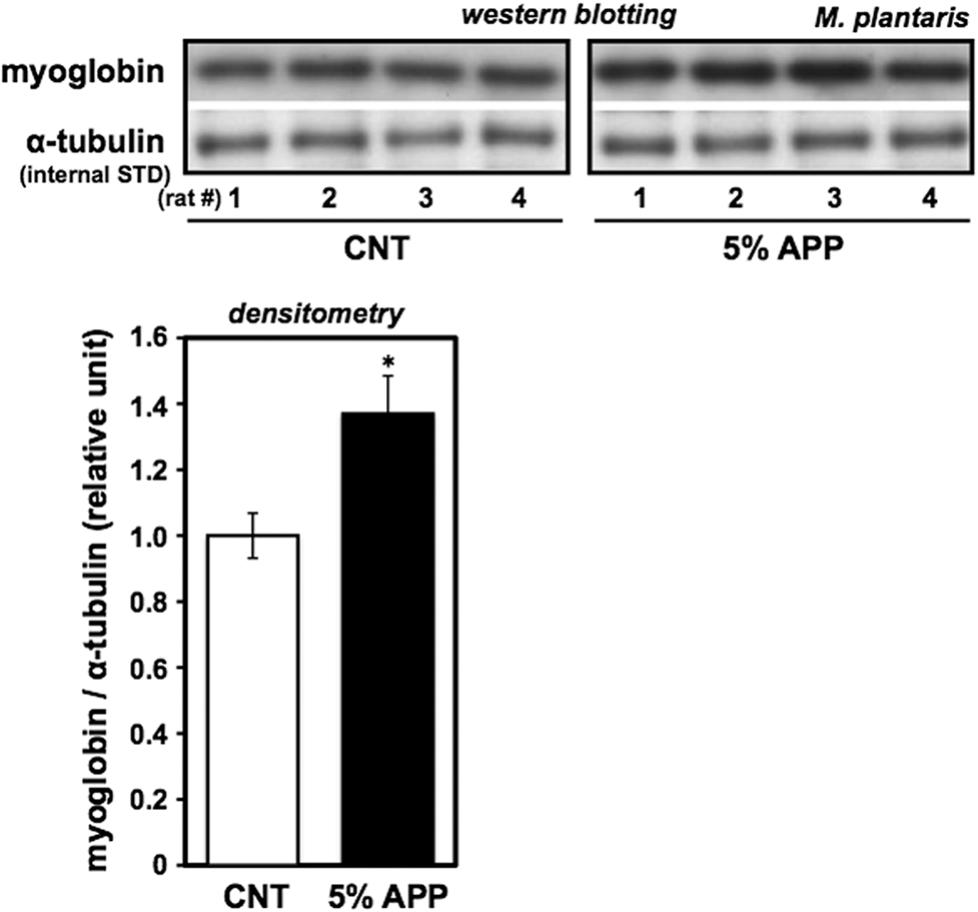
Significant increase in myoglobin protein after 5% APP feeding. Plantaris muscles from control and 5%-APP diet groups (after 8-wk feeding) were analyzed for myoglobin by ECL-western blotting referenced with α-tubulin as an internal standard (upper panel; 4 representative samples from each group). Myoglobin-band intensity was quantified by densitometry and expressed as relative units to the control (lower panel). Data bars present the mean ± SE for nine rats in each group fed with control (open bar) or the 5%-APP diet (closed bar) and a significant difference from control mean at *P* < 0.05 is indicated by (*).

In order to corroborate the observed change in fatigue resistance, the expression of adult MyHC isoforms (MyHC I, IIa, IIx, and IIb, expressed by fiber types from slow to fastest, in that order) and myoglobin, were evaluated for plantaris muscles from the control and 5%-APP pair-fed groups (Figs 2 and 3) at the end of the 8-wk feeding period. Plantaris is a good representative for treatment effects on fiber type distribution, because as a planter flexor muscle, it consists predominantly (over 95%) of IIb and IIx fibers. MyHC isoforms and the relative ratio of each isoform to total MyHC (measured by densitometry of the band intensity) are shown in Fig 2. The relative amount of MyHC IIb was significantly reduced (*P* < 0.05) along with the compensatory increase of MyHC IIx (*P* < 0.01) in the 5%-APP group. The levels of MyHC I and IIa relative to total MyHC did not change in this experiment. Myoglobin protein expression relative to α-tubulin also increased in the 5%-APP group (*P* < 0.05, Fig 3), supporting the finding that fiber-type composition can be altered by a 5%-APP diet for 8 wks. The finding is consistent with the contribution of an increased IIx/IIb ratio to significant improvement of the fatigue resistance of posterior hind limb muscles of male Fischer F344 rats.

### Effects of a 0.5% APP diet

**Fig 4:**
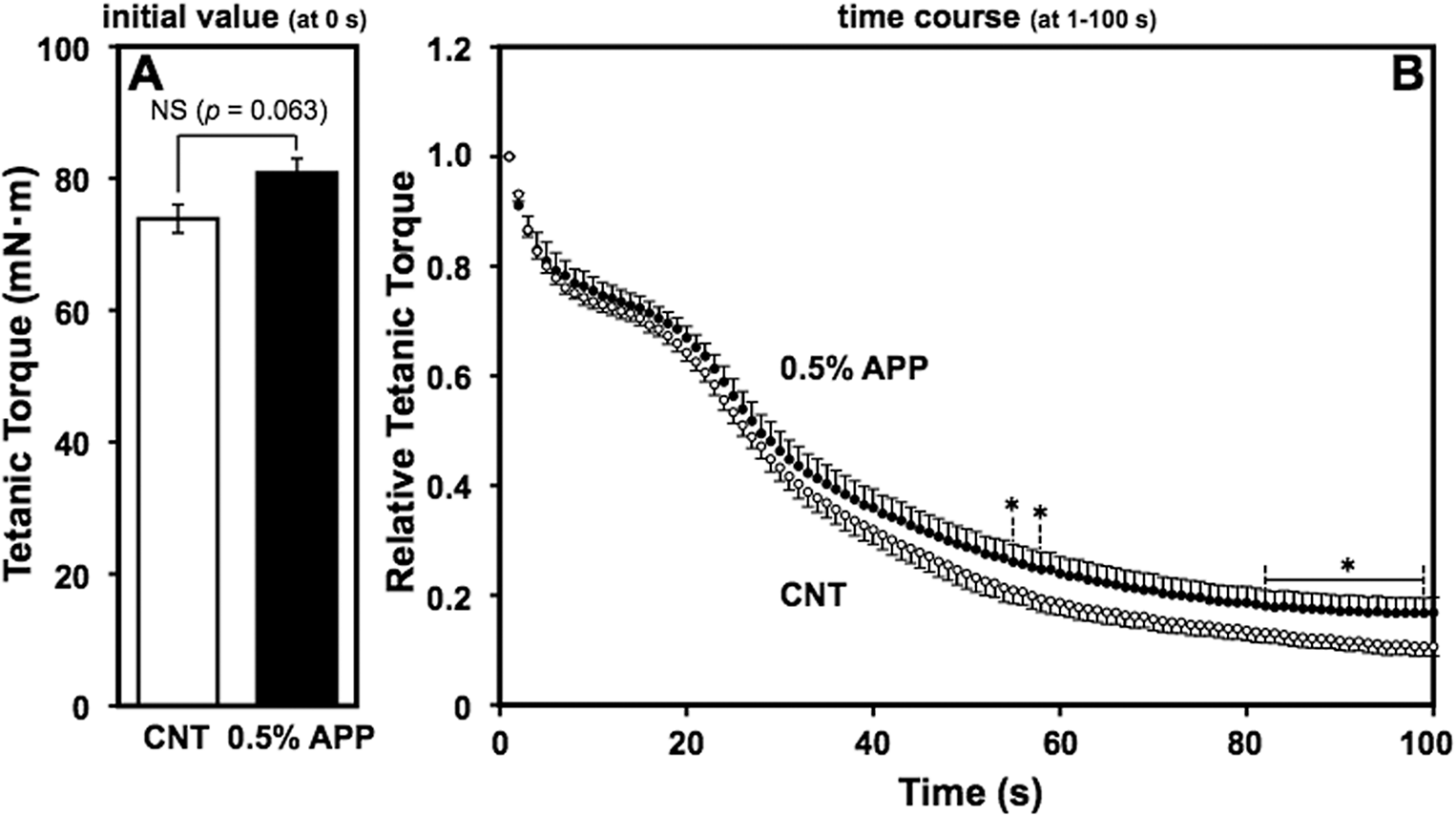
Lower improvement of muscle endurance by 0.5%-APP feeding *vs*. 5% APP. Maximum isometric planter-flexion force torque was measured after 8-wk feeding of the 0.5%-APP or control diet, in the same way as Fig 1. Panel A shows the initial torque values at the first stimulation (at 0-s); panel B shows the time-course of torque values during the 100-s successive stimulation (expressed relative to the initial value). Data points and bars depict the mean ± SE for nine rats in each group fed with a control (open circle and bar) or 0.5%-APP diet (closed circle and bar) and significant differences from control at *P* < 0.05 are indicated by (*). NS, no significant difference (*P* = 0.063).

The above interpretation is supported by results of a supplementary lower-dose APP-diet experiment, in which separate sets of 9-wk-old male Fischer F344 rats were maintained with control and 0.5%-APP (w/w) diet *ad libitum* for 8 wks (*n* = 9/group) and evaluated for fatigue resistance of the calf muscles. Again, growth profiles were comparable between the two groups as monitored by total dietary intake and body weight over time (S3 Fig and S4 Fig panel A) and final organ weights (S4 Fig panel B), along with a trend to reduced adipose tissue weight (S5 Fig panel A). As shown in Fig 4, 0.5% APP-fed rats displayed higher fatigue resistance, revealed by a delayed decline in relative maximum isometric planter-flexion force torque, which was significantly different from torque in control-fed rats at some time-points (55, 58, and 82–99 s) during the 100-s period of electrical stimulation. The change in fatigue resistance was much smaller than for the 5%-APP treatment, and in this group of 0.5% APP-fed rats, there was no significant change in fiber-type composition, suggesting a dose-dependent response to APP. A further experiment of 0.5%-APP *ad libitum* feeding (*n* = 9/group) using male Sprague-Dawley rats with a bigger growth profile than the Fischer F344 strain, demonstrated a significant alteration of fiber-type composition in plantaris and EDL muscles (increased MyHC IIx/IIb and I, IIa/IIx ratios, *P* < 0.05, respectively) (Mizunoya et al., in preparation). Importantly, the experiments also showed that there was no significant difference in the locomotor activity shown as the total distance moved in each 12-h period of light or darkness, between the control and 0.5% APP-diet groups (*n* = 4), assayed after the 6-wk feeding period by a behavioral tests analytical system (O'Hara & Co., Tokyo, Japan) equipped with a video camera (2 frames/sec) and analysis software (Image OF) running on the public domain NIH Image and ImageJ programs (Mizunoya et al., in preparation). Considering these results together, the present study highlights the promising contribution of dietary APP intake to improved endurance based on fiber-type composition in muscle of male rats.

### Discussion on mechanisms of APP activity

The present study identified a significant exercise-independent contribution of APP intake to increasing muscle endurance. However, there are significant gaps in our understanding of the potential mechanism(s) of action of APP in modifying fiber-type composition in rat muscle. We do know that APP is a mixture of polyphenols, including procyanidins, chlorogenic acid, phlorizin, catechin, and quercetin glycosides [17] although its active components are still unknown. In order to address the mechanisms of APP activity, it will be important to know its bioavailability and the levels of APP components in the circulation, although it is generally thought that natural compounds have a low bioavailability resulting in low circulating blood levels. Shoji et al. [27] investigated the absorption of procyanidins which are the most abundant polyphenolic compounds in APP, and hence a primary plausible-candidate for the APP activity. In experiments in 7-wk-old male Wister rats, and using the Porter method and high-performance liquid chromatography/tandem mass spectrometry, results showed that the plasma level of procyanidin (*n* = 8) increased to a maximum (mean ± SE) of 10.2 ± 2.2 μg/ml at 2 h after intragastric injections at a dose of 1000 mg/kg body weight. This effect via direct stomach intubation indicated that procyanidins at least, can be absorbed from the gastrointestinal tract, and that plasma levels may reach a functional range under conditions appropriate for APP administration to animals. The lack of information about the other polyphenols in APP requires similar investigation.

In close association with these considerations, it is worth noting that preliminary results from a study of 5%-APP dietary treatment for 8 wk (S6 Fig) did not demonstrate any alteration of the mRNA expression of PGC1α, an essential element of a transcriptional circuit centered on PGC1α and PPARδ and that subsequently activates the expression program for the slow-fiber phenotype in muscle [7–14]. Similar observations were reported previously by Nakazato et al. [19] in which PPARγ mRNA expression was equivalent in control and 5%-APP diet groups, along with a faint up-regulation of PPARα and δ (about 1.2- and 1.1-fold changes, respectively; *P* < 0.05) relative to glyceraldehyde 3-phosphate dehydrogenase (GAPDH). These findings suggest that APP and/or its metabolites do not mediate the PGC1α/PPARδ-dependent signaling pathway.

Apart from the above PGC1α/PPARδ-dependent signaling pathway, we very recently reported that resident myogenic stem (satellite) cells produce and secrete a large amount of Sema3A protein, and that this occurs exclusively at the early differentiation phase and stimulates the formation of slow, type-I fibers in adult mice and rats [22, 28–36]. Notably, a subsequent *in vitro* study suggested that supplementation of APP (500 ng/ml) or chlorogenic acid (10 ng/ml) also up-regulated the expression of MyHC-I and the up-stream signaling molecules, myogenin and myocyte-specific enhancer factor 2D (MEF2D) in primary cultures of differentiating myoblasts. By comparison, other major polyphenols found in APP (procyanidin B1, B2, phloridzin, and catechin) in a range of 10–1000 ng/ml did not induce these effects (Suzuki et al., manuscript in preparation). However, the novel Sema3A-myogenin-MEF2D-slow MyHC signaling pathway is still a working hypothesis for slow-fiber formation during muscle regeneration after injury, and there is no direct evidence that chlorogenic acid associates with a Sema3A membrane receptor to induce slow MyHC expression. Fewer than 10% of the satellite cell population in un-injured muscle of adult animals is mitotically active and can proliferate, differentiate, and fuse to existing or new myofibers to help maintain muscle mass, structure and function. One possibility is that the chlorogenic acid component in APP may act as an agonist for Sema3A, in stimulating or enhancing slow-fiber generation by satellite cell-derived myoblasts in un-injured muscle. In this hypothetical view, the outcome may only be detectable after a longer period than required for muscle regeneration as the proliferative population of satellite cells is so small without muscle damage or exercise. Consistent with this idea, when plantaris muscle was examined after the 8-wk feeding period of 5% APP, fibers showed weakly positive staining for MyHC I (S7 Fig, light-brown fibers indicated by closed arrowheads), suggesting a fast-to-slow remodeling of fibers in response to the APP diet, as it was not observed in sections of control muscle. These observations await further study of the molecular mechanisms by which dietary polyphenol intake alters the fiber-type composition in young-adult mice and rats.

Finally, it may be worth noting that treatment of 4–8 wk-old male C57BL/6J mice with 0.4% (w/w) resveratrol (3,4',5-trihydroxy-trans-stilbene, a natural polyphenolic compound mainly found in grape skins, peanuts, and red wine [37]), significantly increases their aerobic capacity, as evidenced by an increased running time and higher oxygen consumption by muscle fibers *via* activation of SIRT1 and PGC1α [38]. Such effects are similar to the fatigue-resistant effect of tea polyphenol [15, 16] and were anticipated in a report by Nakazato et al. [39] that showed a 5%-APP intake prevented muscle injury induced by lengthening contractions in 10-wk-old male Wistar rats, possibly by a fast-to-slow fiber-type transition. We have also reported that a diet of 15% (w/w) fish oil in AIN-93G (in a 4-wk, pair-feeding study) up-regulates expression of MyHC IIx and a number of metabolic genes (mitochondrial uncoupling protein 3, pyruvate dehydrogenase kinase 4, and porin) in EDL muscle of 7-wk-old male Wistar rats (see Ref. [40] for more detail). Considering the earlier pioneering observations of APP and this recent report, we can speculate that the myofiber-type composition in fast muscle can be shifted independent of exercise, toward a more intermediate or slow-fiber distribution by a few dietary components, including catechins, resveratrol, and n-3 polyunsaturated fatty acids (PUFA) in addition to the APP concerned in this study. While their modes of action are still unclear and likely to be differential, their similar outcome, centered on a significant increase in the percentage of type-I fibers and induction of an oxidative metabolic phenotype in muscle (*P* < 0.05), was also observed after very high doses of vitamin B_3_ (niacin; 750 mg/kg diet or 1 g/day by drenching) over 3–4 wks, to 11-wk-old growing male pigs and sheep [41, 42]. Very recently, Terao and Nikawa and their colleagues demonstrated that muscle atrophy from disuse due to tail-suspension or denervation can be prevented by other polyphenols including quercetin (3,5,7,3′,4′-pentahydroxyflavone, by intramuscular injection of 2.5 pmol at 24-h intervals) [43] and 8-prenylnaringenin (a metabolite of isoxanthohumol, 5.6 mmol/kg diet) [44] and by soy glycinin protein (10–20% in diet) [45] in 5-7-wk-old male C57/BL6 mice. These treatments also suppressed the expression of key ubiquitin ligases involved in muscle atrophy, through the protection of Akt phosphorylation, suggesting that as a possible mode of action for these compounds that maintain muscle mass [43–45]. Considering that the atrophy of disuse/denervation occurs preferentially in slow fibers [46], these compounds of vegetable origin could work selectively on slow fibers so they resist atrophy. An appropriate combination of functional food ingredients such as atrophy-preventive molecules (quercetin, 8-prenylnaringenin, and glycinin) and endurance enhancers (catechins, resveratrol, APP, and PUFA) all of vegetable origin except for PUFA, seems to be a promising way to develop and enhance the pharmacological and physiological toolkit to promote human health and performance and for medical therapy of muscle atrophy.

## Conclusion

This pair-feeding study reports that an 8-wk intake of 5% APP significantly up-regulates two important elements related to slow fibers: the MyHC type IIx/IIb ratio and the level of myoglobin protein, in plantaris muscle of 9-wk-old male Fischer F344 rats. Findings may account for increased fatigue resistance demonstrated by a significantly slower decline in the maximum isometric force torque generated by planter-flexor contraction stimulated *via* the tibial nerve. Additionally, the smaller level of fatigue resistance displayed by rats fed with only 0.5% APP supports the notion of an APP-dose dependency for the fast-to-slow alteration of fiber-type composition. The mechanism(s) underlying the action of APP, including a cell-membrane receptor for active components of APP and/or their metabolites, are still unclear. Since fiber-type distribution in muscle is responsible for its sensory, contractile, and metabolic properties, this new information about dietary manipulation of fiber-type composition reveals the potential of a novel strategy to improve animal-meat production, human athletic performance, and medical therapy for age-related muscle atrophy.

## Supporting Information

S1 ARRIVE Checklist The Animal Research: Reporting In Vivo Experiments (ARRIVE) guidelines checklist. (PDF)

S1 Table Composition of experimental diets. (PDF)

S1 Fig Comparative growth profiles of animals fed with control and 5% APP diets. Total diet intake (panel A) and body weight (panel B) were measured throughout the 8-wk pair-feeding of control (open squares and bars) and 5% APP diets (closed circles and bars). Immediately after anesthesia and exsanguination, the liver, kidney, spleen, and heart were collected and weighed (panel C; expressed as g/100 g body weight). Data points and bars depict the means ± SEs for nine rats per treatment group and there were no significant differences from control at *P* < 0.05 in all parameters measured at the end of the experimental period, except for heart weight as indicated by (*). NS, no significant difference.
(PDF)

S2 Fig Weight of adipose tissue and muscle for animals fed with control and 5% APP diets. Adipose tissues including epididymal, perinephric, mesenteric fats, and brown adipose tissue were collected from rats fed with control (open bars) and 5% APP diets (closed bars) at the end of the 8-wk experimental period and weighed (panel A; expressed as g/100 g body weight). Muscles from the lower hind limbs (soleus, plantaris, tibialis anterior, gastrocnemius, and EDL) were also weighed (panel B; total weight of right and left-sided muscles, expressed as g/100 g body weight). Panel C, total protein content in plantaris muscle by a Dumas combustion method (expressed as % dry weight). Bars depict the mean ± SE for *n* = 9 rats per treatment group; significant differences from control at *P* < 0.05 and *P* < 0.01 are indicated by (*) and (**), respectively. NS, no significant difference. These profiles provide evidence for the reliability of the pair-feeding conditions.
(PDF)

S3 Fig Time-course of the total diet intake and body weight in control diet and 0.5% APP diet groups. Male Fischer F344 rats (9-wks-old) were fed with a control (open squares) or 0.5% (w/w) APP diet (closed circles) *ad libitum* for 8 wks. The time-course of total food intake (panel A) and body weight (panel B) were depicted. See S4 Fig for more information.
(PDF)

S4 Fig Comparative growth profiles of animals fed with control or 0.5% APP diets *ad libitum*. Male Fischer F344 rats (9-wks-old) were fed with a control (open bars) or 0.5% (w/w) APP diet (closed bars) *ad libitum* for 8 wks; total diet intake and body weight (panel A) and internal organ weights (panel B) were measured at the end of the experimental period as described in S1 Fig. Data bars depict the mean ± SE for nine rats per treatment group and there were no significant differences (NS) from control at *P* < 0.05 for all parameters measured. The time-course of total food intake and body weight are shown in S3 Fig.
(PDF)

S5 Fig Adipose tissue and muscle weight for animals fed with control or 0.5%-APP diets. Adipose tissues (epididymal fat, perinephric fat, mesenteric fat, and brown adipose tissue) (panel A) and muscles from lower hind limbs (soleus, plantaris, tibialis anterior, gastrocnemius, and EDL) (panel B) of rats fed with control (open bars) or a 0.5%-APP diet (closed bars) were weighed at the end of the 8-wk treatment period, as described in S2 Fig Panel C, total protein content in plantaris muscle was also measured as described in S2 Fig panel C. Data bars present the mean ± SE for nine rats per treatment group and significant differences from control at *P* < 0.05 and *P* < 0.01 are indicated by (*) and (**), respectively. ^#^, *P* < 0.1. NS, no significant difference.
(PDF)

S6 Fig Comparable PGC1α-mRNA expression in plantaris muscles from control and 5% APP-diet groups (after 8-wk feeding). PGC1α mRNA expression was monitored after 8-wks feeding by real-time RT-qPCR run under the TaqMan probe detection format, standardized to the expression of HPRT. NS, no significant difference.
(PDF)

S7 Fig Immunohistochemistry of slow MyHC-positive fibers in plantaris muscles from control and 0.5% APP-diet groups (after 8-wk feeding). Cryosections of plantaris muscle were immunostained with monoclonal anti-MyHC type-I and HRPO-labeled secondary antibodies followed by colorization with DAB substrate and counter-staining with hematoxylin. Consistent with the quantitative analysis of relative MyHC isoform content by our SDS-PAGE system (see Fig 2), slow fibers were not prevalent in plantaris muscle; however, areas of slow fibers were identified (upper panel) and magnified views of the boxed areas are shown in the second row. Note that slow MyHC-positive myofibers (representative fibers are indicated by open arrowheads) are clearly observed along with the presence of weakly-stained fibers (indicated by closed arrowheads in APP-fed group) that are easily distinguished from negative fibers (fast fibers).
(PDF)
