# Supplementary material for: Improvement of Endurance Based on Muscle Fiber-Type Composition by Treatment with Dietary Apple Polyphenols in Rats
Source: PLoS One. 2015 Jul 29;10(7):e0134303. doi: 10.1371/journal.pone.0134303 (PMC4519157; doi:10.1371/journal.pone.0134303)
Supplement: S1 Table — (PDF) [file pone.0134303.s002.pdf]

S1 Table. Composition of experimental diets[a]

Ingredient (g / 100 g diet)	Groups		
	CNT	5% APP	0.5% APP
**cornstarch**[b]	39.6486	34.6486	39.1486
**α-cornstarch**[b]	13.2	13.2	13.2
**casein**[b]	20.0	20.0	20.0
**sucrose**[b]	10.0	10.0	10.0
**soybean oil**[b]	7.00	7.00	7.00
**cellulose**[b]	5.00	5.00	5.00
**AIN-93G-MX mineral mix**[b]	3.50	3.50	3.50
**AIN-93-VX vitamin mix**[b]	1.00	1.00	1.00
**L-cystine**[c]	0.30	0.30	0.30
**choline bitartrate**[c]	0.25	0.25	0.25
**L-ascorbic acid (vitamin C)**[c]	0.10	0.10	0.10
**t-butylhydroquinone**[d]	0.0014	0.0014	0.0014
**apple polyphenol (APP)**[e]	0.00	5.00	0.50

[a] Based on the AIN-93G formulation containing additional L-ascorbic acid, which is not included in the AIN-93-VX vitamin mix.

[b-d] Purchased from Oriental Yeast (Tokyo, Japan), Sigma (St. Louis, MO, USA), and Wako Pure Chemical Industries (Osaka, Japan), respectively.

[e] Supplied from Fundamental Research Laboratory, Asahi Breweries (Moriya, Ibaraki, Japan).
