# Supplementary figures and images for: Improvement of Endurance Based on Muscle Fiber-Type Composition by Treatment with Dietary Apple Polyphenols in Rats
Source: PLoS One. 2015 Jul 29;10(7):e0134303. doi: 10.1371/journal.pone.0134303 (PMC4519157; doi:10.1371/journal.pone.0134303)

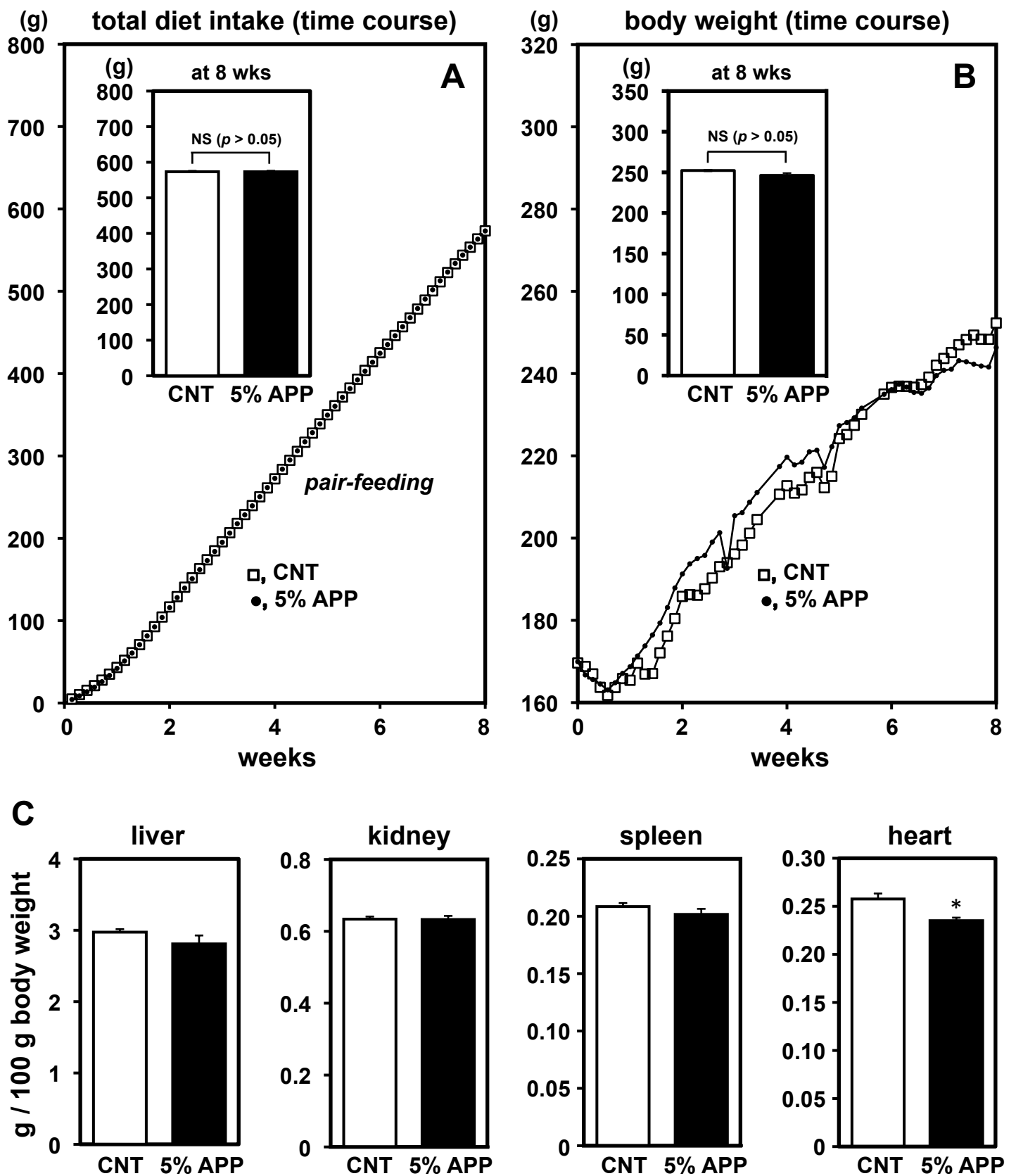

**S1 Fig., Mizunoya et al., PLoS ONE**

Supplement: S1 Fig — Total diet intake (panel A) and body weight (panel B) were measured throughout the 8-wk pair-feeding of control (open squares and bars) and 5% APP diets (closed circles and bars). Immediately after anesthesia and exsanguination, the liver, kidney, spleen, and heart were collected and weighed (panel C; expressed as g/100 g body weight). Data points and bars depict the means ± SEs for nine rats per treatment group and there were no significant differences from control at P < 0.05 in all parameters measured at the end of the experimental period, except for heart weight as indicated by (*). NS, no significant difference. (PDF) [file pone.0134303.s003.pdf]

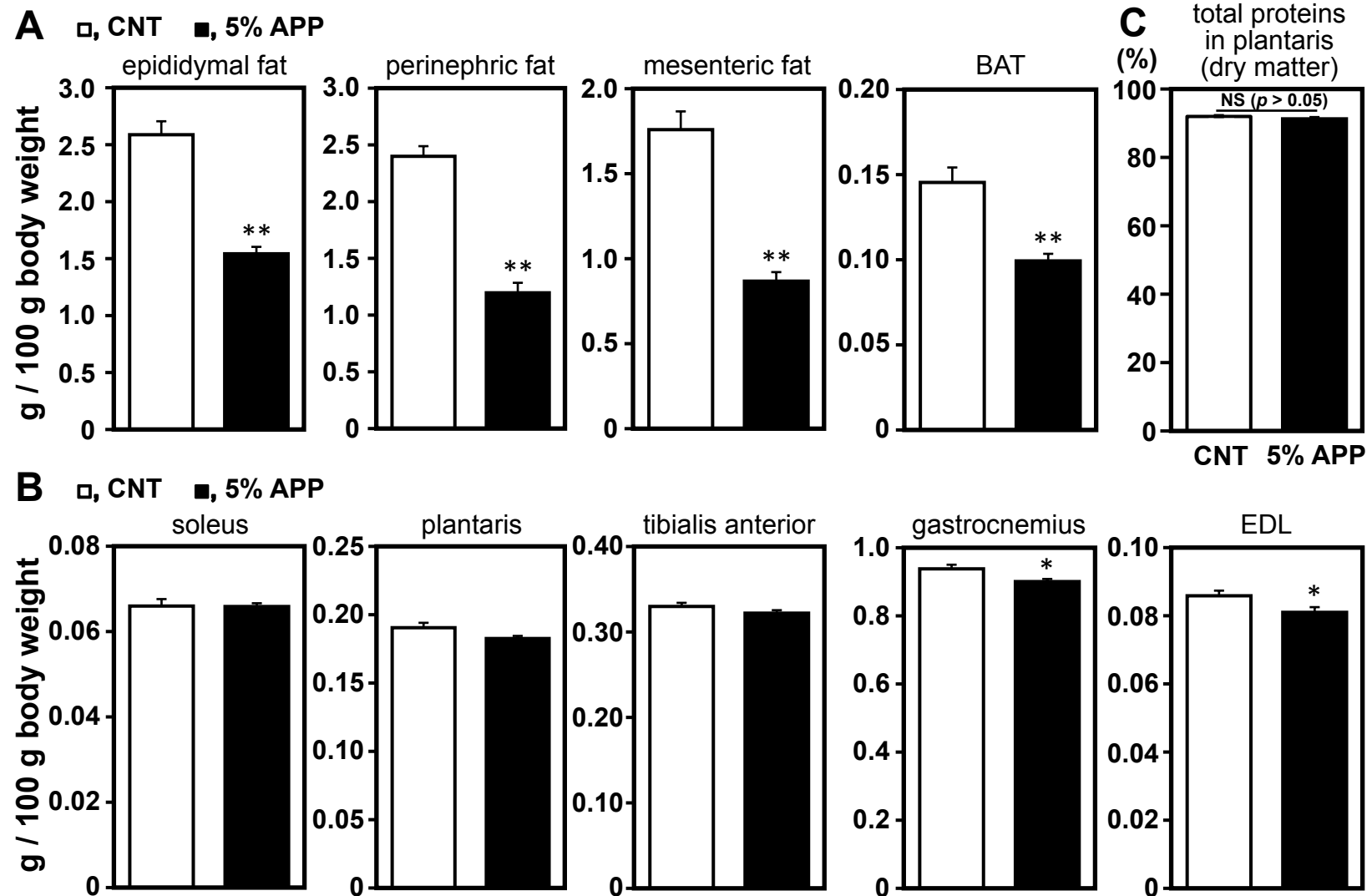

S2 Fig., Mizunoya *et al.*, PLoS ONE

Supplement: S2 Fig — Adipose tissues including epididymal, perinephric, mesenteric fats, and brown adipose tissue were collected from rats fed with control (open bars) and 5% APP diets (closed bars) at the end of the 8-wk experimental period and weighed (panel A; expressed as g/100 g body weight). Muscles from the lower hind limbs (soleus, plantaris, tibialis anterior, gastrocnemius, and EDL) were also weighed (panel B; total weight of right and left-sided muscles, expressed as g/100 g body weight). Panel C, total protein content in plantaris muscle by a Dumas combustion method (expressed as % dry weight). Bars depict the mean ± SE for n = 9 rats per treatment group; significant differences from control at P < 0.05 and P < 0.01 are indicated by (*) and (**), respectively. NS, no significant difference. These profiles provide evidence for the reliability of the pair-feeding conditions. (PDF) [file pone.0134303.s004.pdf]

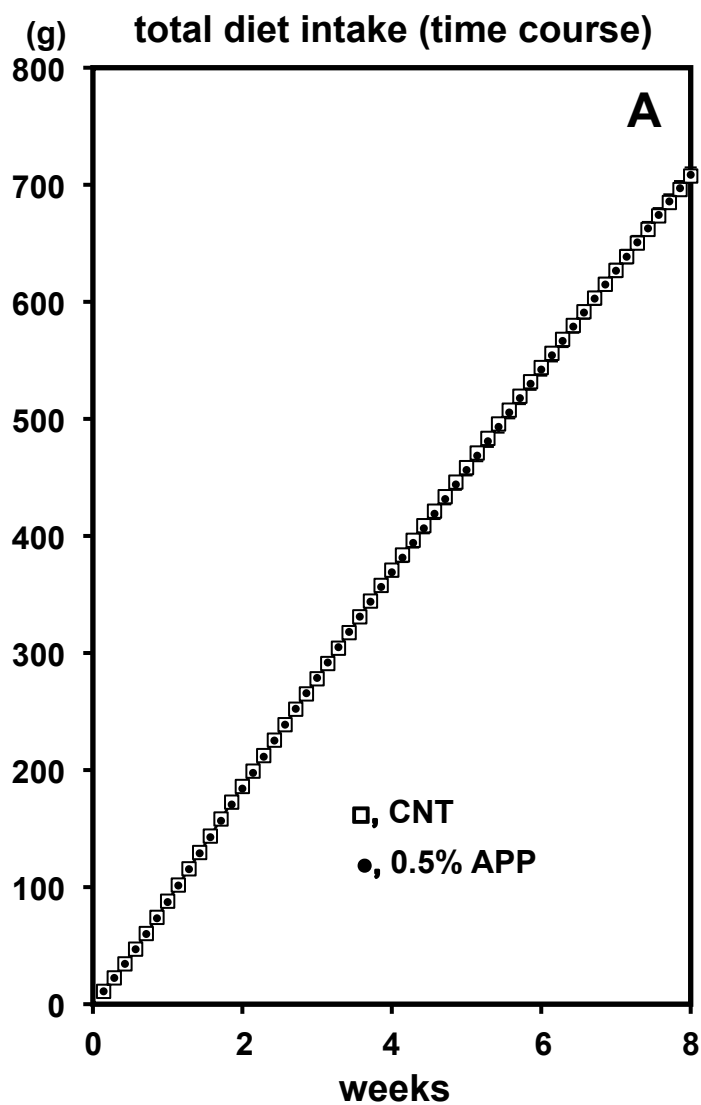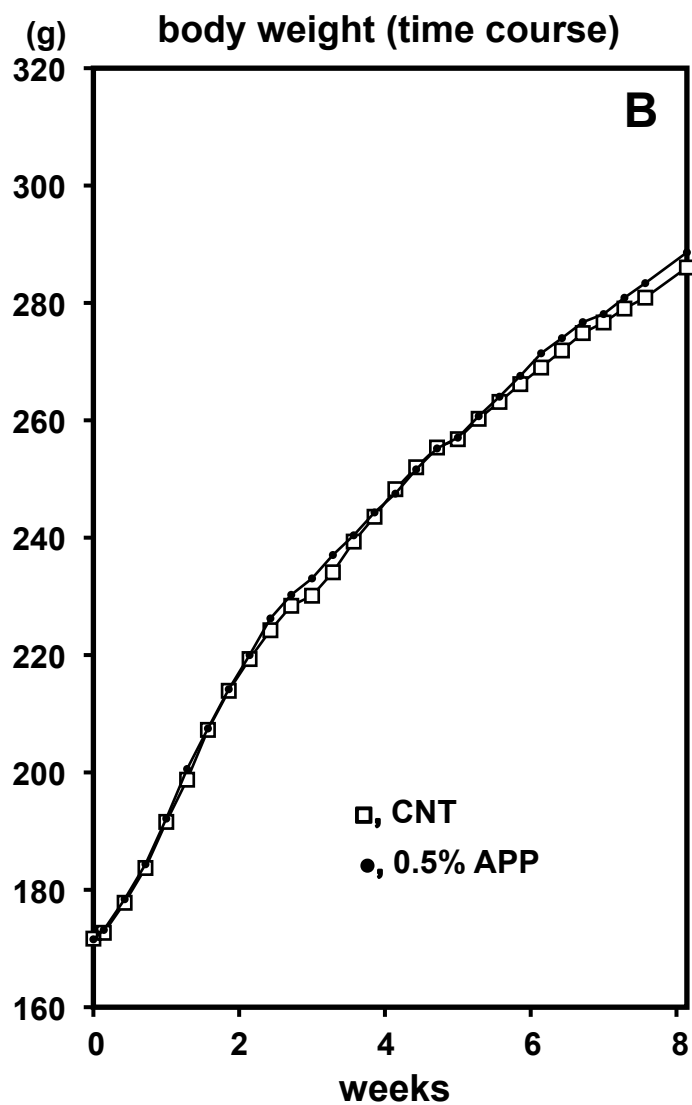

**S3 Fig., Mizunoya *et al.*, *PLoS ONE***

Supplement: S3 Fig — Male Fischer F344 rats (9-wks-old) were fed with a control (open squares) or 0.5% (w/w) APP diet (closed circles) ad libitum for 8 wks. The time-course of total food intake (panel A) and body weight (panel B) were depicted. See S4 Fig for more information. (PDF) [file pone.0134303.s005.pdf]

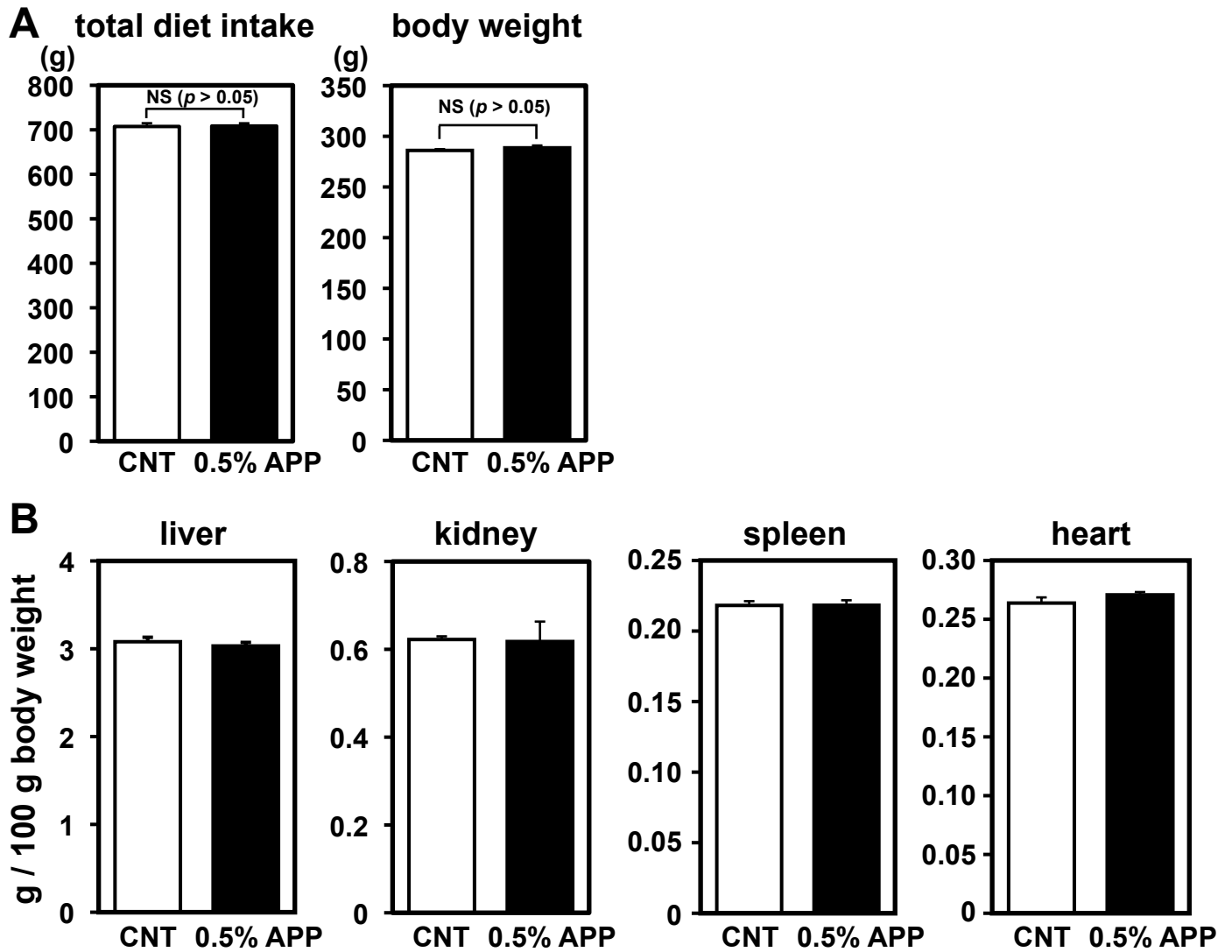

**S4 Fig., Mizunoya *et al.*, PLoS ONE**

Supplement: S4 Fig — Male Fischer F344 rats (9-wks-old) were fed with a control (open bars) or 0.5% (w/w) APP diet (closed bars) ad libitum for 8 wks; total diet intake and body weight (panel A) and internal organ weights (panel B) were measured at the end of the experimental period as described in S1 Fig. Data bars depict the mean ± SE for nine rats per treatment group and there were no significant differences (NS) from control at P < 0.05 for all parameters measured. The time-course of total food intake and body weight are shown in S3 Fig. (PDF) [file pone.0134303.s006.pdf]

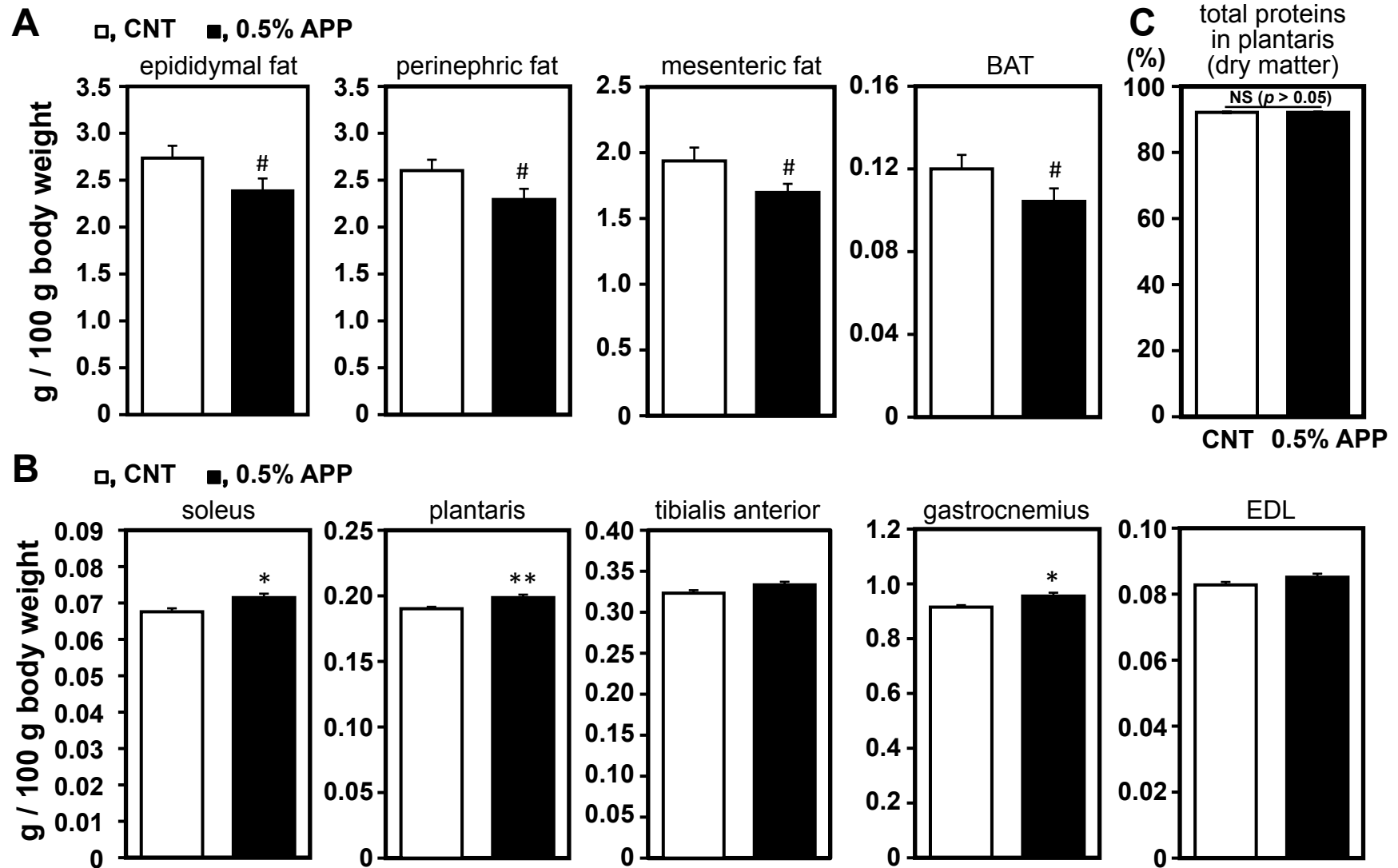

S5 Fig., Mizunoya *et al.*, PLoS ONE

Supplement: S5 Fig — Adipose tissues (epididymal fat, perinephric fat, mesenteric fat, and brown adipose tissue) (panel A) and muscles from lower hind limbs (soleus, plantaris, tibialis anterior, gastrocnemius, and EDL) (panel B) of rats fed with control (open bars) or a 0.5%-APP diet (closed bars) were weighed at the end of the 8-wk treatment period, as described in S2 Fig Panel C, total protein content in plantaris muscle was also measured as described in S2 Fig panel C. Data bars present the mean ± SE for nine rats per treatment group and significant differences from control at P < 0.05 and P < 0.01 are indicated by (*) and (**), respectively. #, P < 0.1. NS, no significant difference. (PDF) [file pone.0134303.s007.pdf]

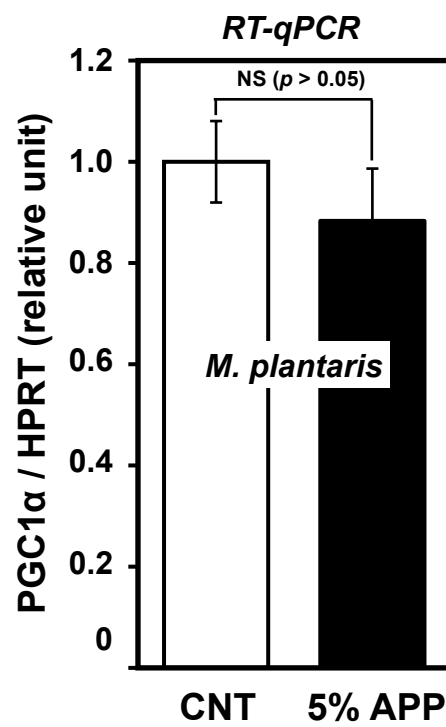

**S6 Fig., Mizunoya *et al.*, PLoS ONE**

Supplement: S6 Fig — PGC1α mRNA expression was monitored after 8-wks feeding by real-time RT-qPCR run under the TaqMan probe detection format, standardized to the expression of HPRT. NS, no significant difference. (PDF) [file pone.0134303.s008.pdf]

**CNT**

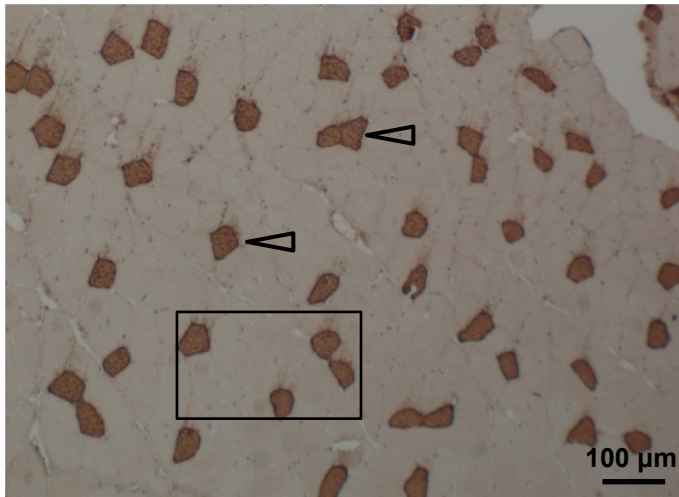

**0.5% APP**

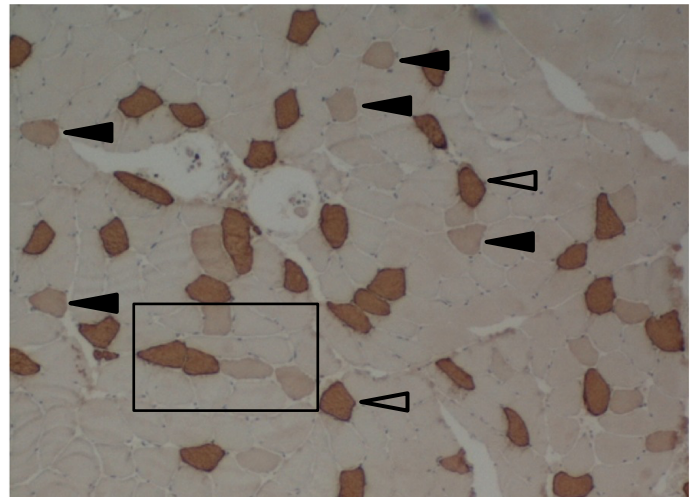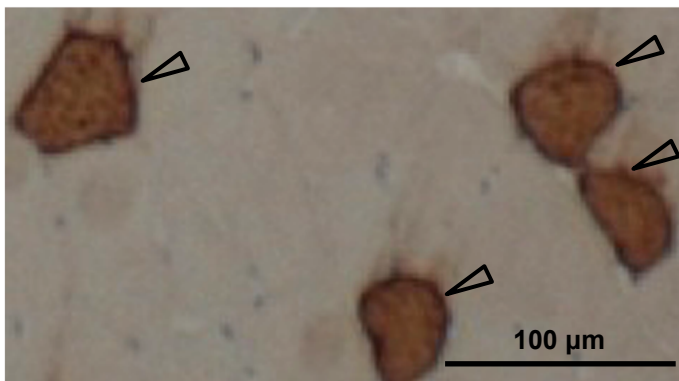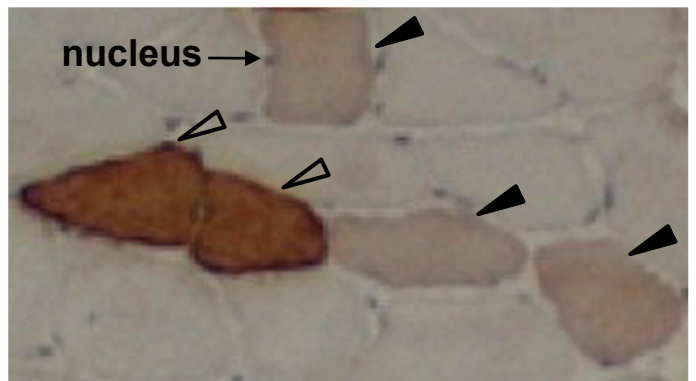

***M. plantaris*** (anti-MyHC I / hematoxylin staining)

Supplement: S7 Fig — Cryosections of plantaris muscle were immunostained with monoclonal anti-MyHC type-I and HRPO-labeled secondary antibodies followed by colorization with DAB substrate and counter-staining with hematoxylin. Consistent with the quantitative analysis of relative MyHC isoform content by our SDS-PAGE system (see Fig 2), slow fibers were not prevalent in plantaris muscle; however, areas of slow fibers were identified (upper panel) and magnified views of the boxed areas are shown in the second row. Note that slow MyHC-positive myofibers (representative fibers are indicated by open arrowheads) are clearly observed along with the presence of weakly-stained fibers (indicated by closed arrowheads in APP-fed group) that are easily distinguished from negative fibers (fast fibers). (PDF) [file pone.0134303.s009.pdf]
